# Supplementary material for: DMRforPairs: identifying Differentially Methylated Regions between unique samples using array based methylation profiles
Source: BMC Bioinformatics. 2014 May 15;15:141. doi: 10.1186/1471-2105-15-141 (PMC4046028; doi:10.1186/1471-2105-15-141)
Supplement: Additional file 2 — DMRforPairs output for the comparison of A431-MCF7 and NA17018-NA17105. Please start from the HTML files in each folder. Available via the BMC Bioinformatics website. [file 1471-2105-15-141-S2.zip › 1394847754114233_MOESM2_ESM/A431_MCF7/figures/37392.pdf]

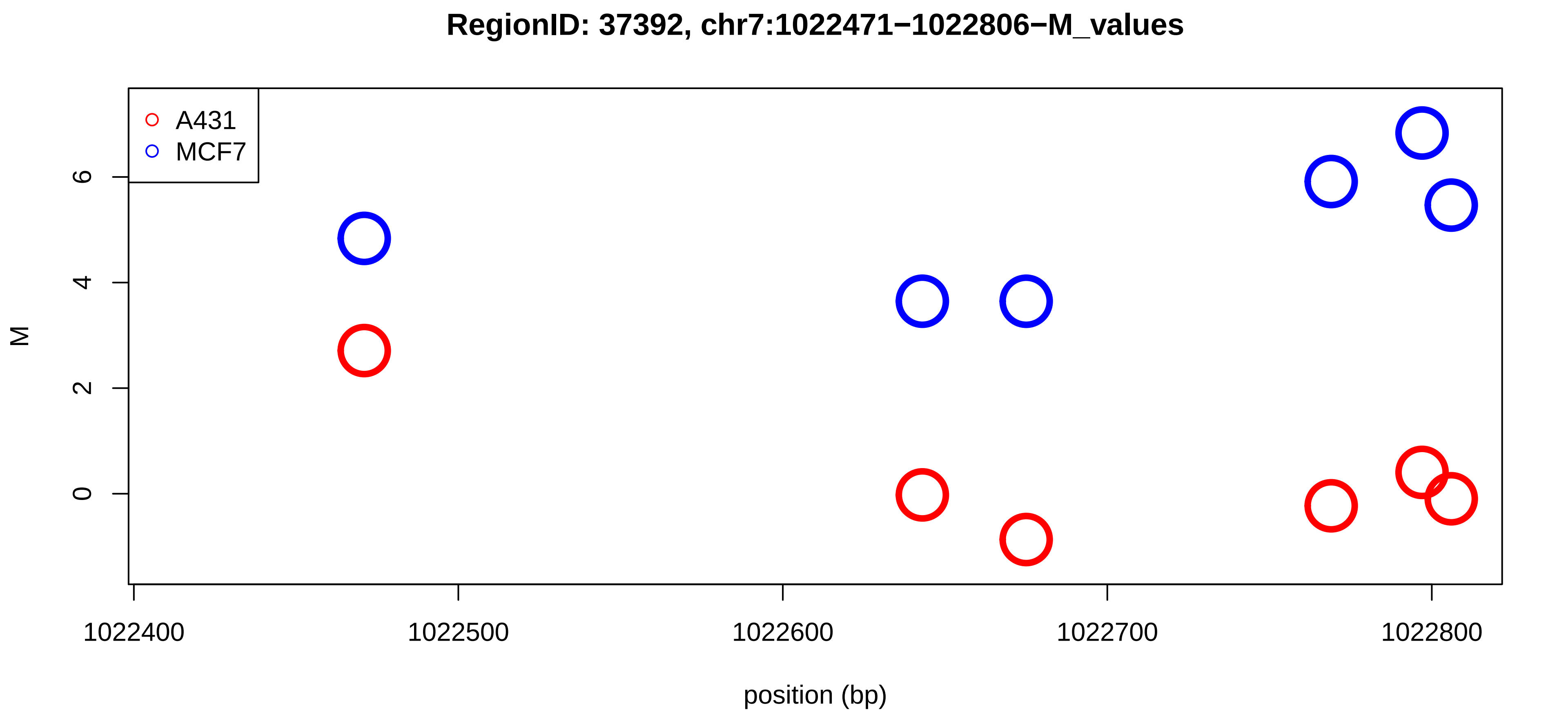
RegionID: 37392, chr7:1022471−1022806−M_values
A431
MCF7
M
0
2
4
6
1022400
1022500
1022600
1022700
1022800
position (bp)

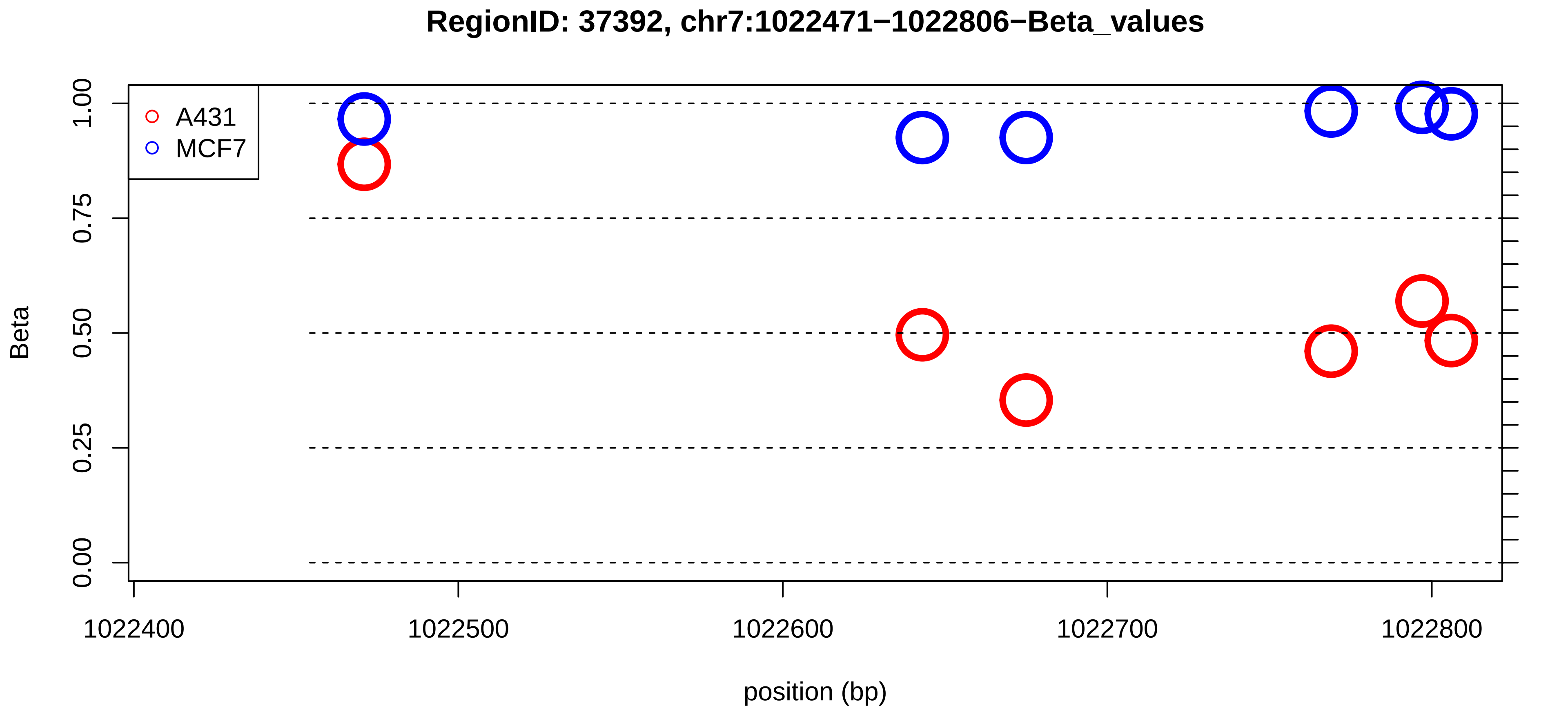
RegionID: 37392, chr7:1022471−1022806−Beta_values
A431
MCF7
Beta
0.00
0.25
0.50
0.75
1.00
1022400
1022500
1022600
1022700
1022800
position (bp)

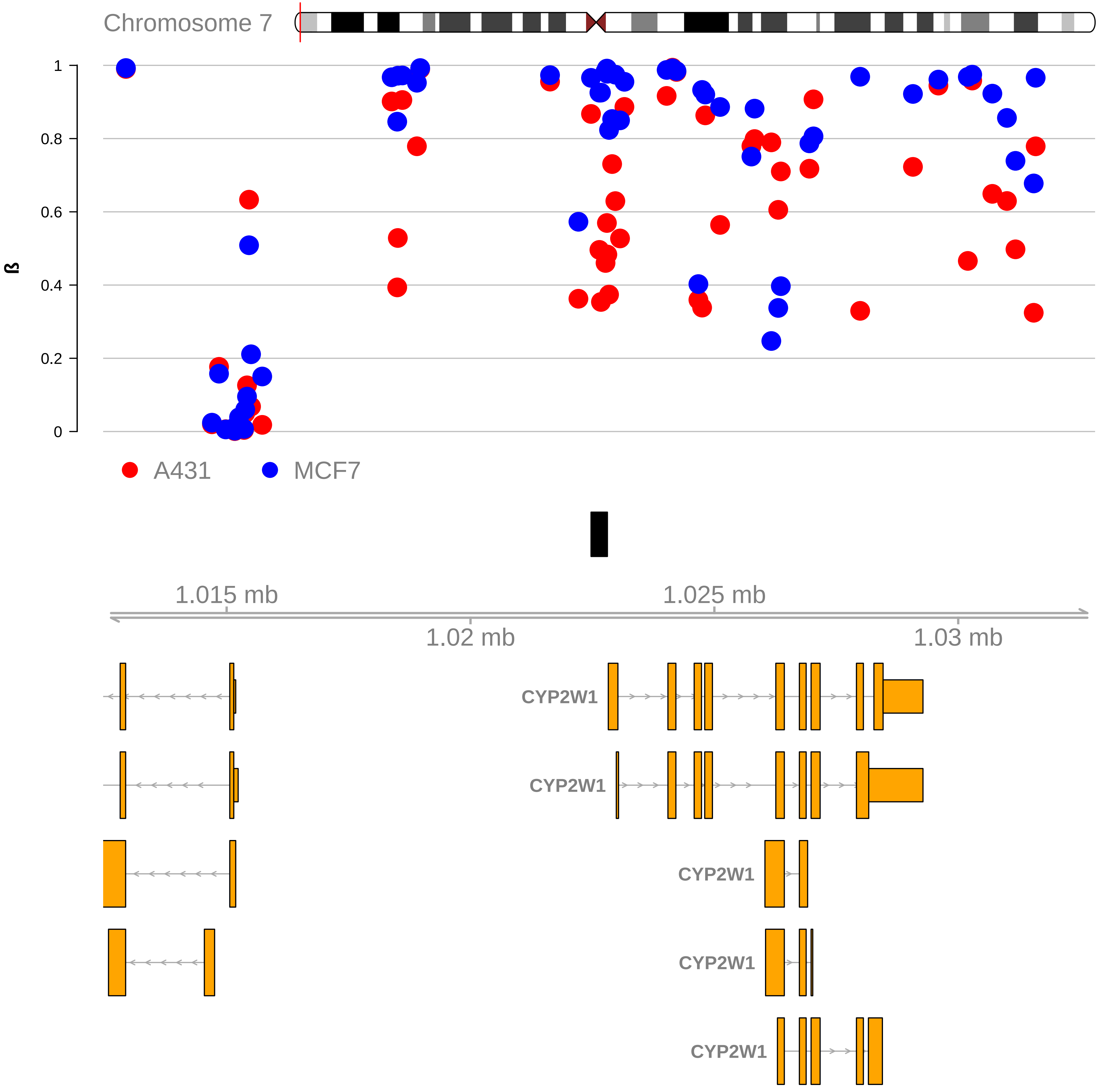

Chromosome 7
ß
1
0.8
0.6
0.4
0.2
0
A431
MCF7
1.015 mb
1.025 mb
1.02 mb
1.03 mb
CYP2W1
CYP2W1
CYP2W1
CYP2W1
CYP2W1
